# A Case of Spontaneous Ruptured Solid Pseudopapillary Tumor of Pancreas Resected by Laparoscopic Surgery

**DOI:** 10.1155/2013/953240

**Published:** 2013-04-30

**Authors:** Susumu Takamatsu, Hiroto Nagano, Shunroh Ohtsukasa, Yasuyuki Kawachi, Hiroshi Maruyama

**Affiliations:** Department of Surgery, Musashino Red Cross Hospital, 1-26-1 Kyounan-cho, Musashino-shi, Tokyo 180-8610, Japan

## Abstract

Solid pseudopapillary tumor (SPT) is an uncommon neoplasm of the pancreas. A rare case of spontaneous rupture of SPT is reported. A 13-year-old female felt acute abdominal pain without blunt abdominal trauma. Enhanced computed tomography (CT) revealed a tumor in the pancreas tail with fluid collection around it. The tumor was diagnosed as SPT with hemoperitoneum associated with spontaneous rupture. The bleeding was stopped conservatively and she was referred for surgery at three months after the rupture. At that time, CT revealed a tumor 4 cm in diameter, which protruded from pancreas tail without distant metastases. Since peritoneal dissemination was not seen on intraoperative exploration, laparoscopic enucleation was performed. Pathologically, the tumor was diagnosed as SPT with rupture of the capsule of tumor, and complete resection was confirmed. The patient has been followed up for two years, and she is alive without recurrence.

## 1. Introduction

Solid pseudopapillary tumor (SPT) of the pancreas was first described by Frantz in 1959 [1]. SPT is regarded as an uncommon low grade malignant tumor accounting for 1-2% of exocrine pancreatic tumors, usually affecting young females [2–4]. The clinical presentations of SPT have been various and not specific. SPT was discovered by rupture and hemoperitoneum which was considered to be rare. In the literature, most of the ruptures of SPT were associated with the blunt abdominal trauma [5–7], while the spontaneous rupture seemed to be quite uncommon. We report a rare case of a spontaneous ruptured SPT which was resected completely by elective laparoscopic surgery.

## 2. Case Report

**Figure 1:**
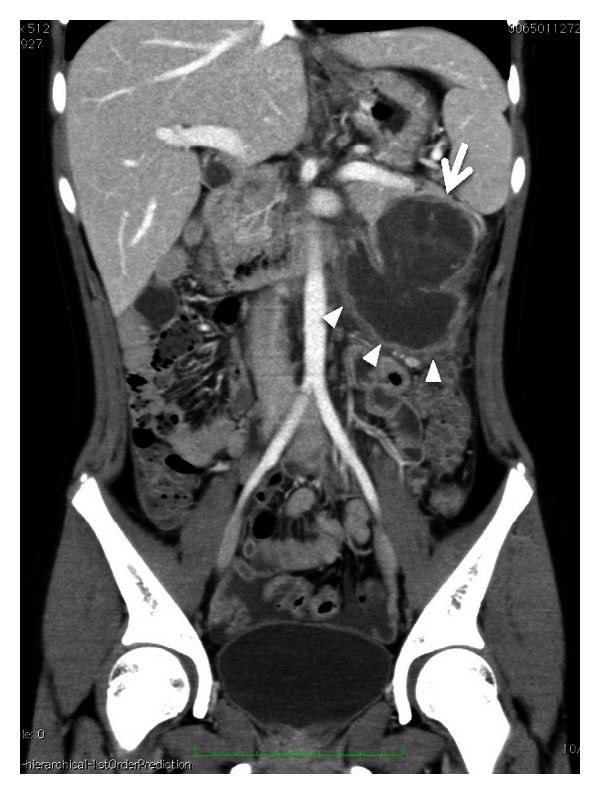
Urgent CT of acute abdomen. A cystic tumor is shown (arrow) in the pancreatic tail and fluid collection (arrowhead) below it. CT also revealed fluid retention in the pelvis. These findings suggested the rupture of tumor and hemoperitoneum.

**Figure 2:**
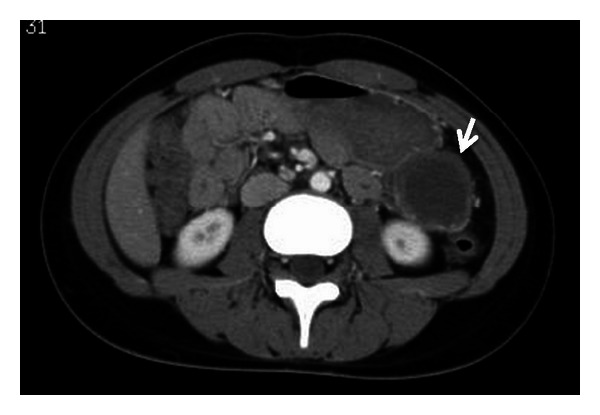
CT at three months after rupturing tumor. A cystic tumor is shown (arrow), protruding caudally from the pancreatic tail. The fluid around tumor had disappeared.

**Figure 3:**
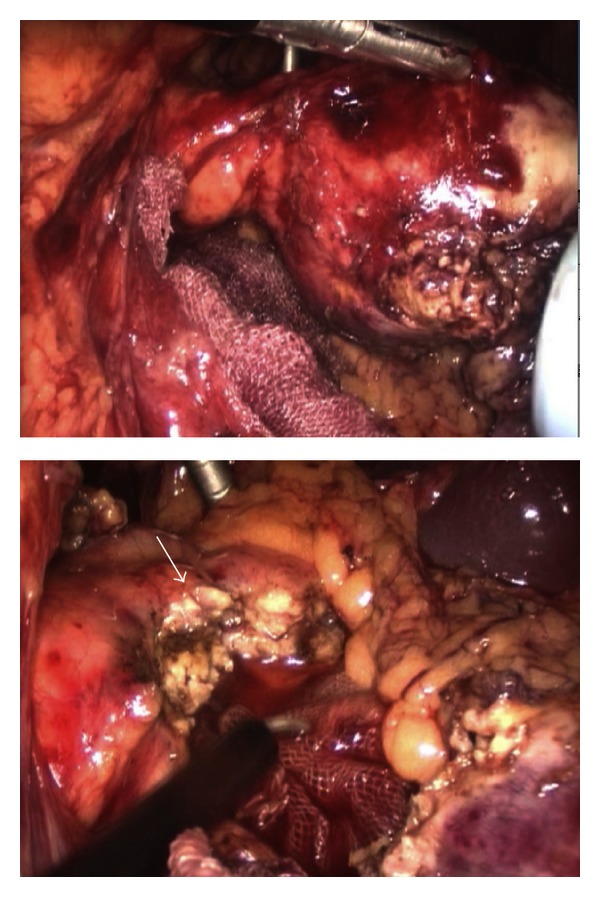
Intraoperative findings and surgical procedure. Above: tumor was grasped by forceps. Tumor was confirmed to be well demarcated and protruded from the pancreas tail. Peritoneal dissemination was not seen on laparoscopic exploration. Below: the tumor was resected with small amount of pancreatic parenchyma. Pancreatic parenchyma was divided by using laparoscopic coagulation shears. Arrow shows the cut surface of the pancreas.

**Figure 4:**
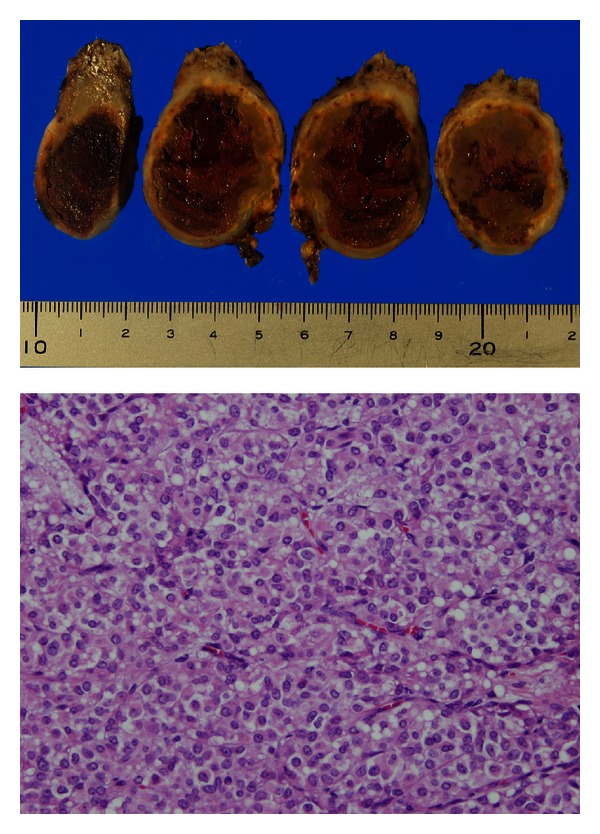
Resected specimen. Above: macroscopically, the tumor contained the necrosis after bleeding. However, there was no apparent cystic part in the tumor. Below (Hematoxylin and Eosin stain ×400): showing solid part of the tumor composed of sheet of tumor cells with ovoid nuclei and with eosinophilic and clear vacuolar cytoplasm. Mitosis was seldom seen.

The patient was a healthy 13-year-old female, who suddenly felt abdominal pain. She was taken to another hospital by ambulance, and the enhanced abdominal computed tomography (CT) revealed a 5 cm diameter cystic tumor in the pancreatic tail with fluid collection around it (Figure 1). The CT results indicated her acute abdominal pain was due to the hemoperitoneum caused by rupture of the pancreatic tumor. Because she had no history of blunt abdominal trauma, the tumor was considered to have ruptured spontaneously. The pancreatic tumor was diagnosed as a SPT of the pancreas from the finding on the CT combined with her age and gender. Since the bleeding stopped by conservative therapy, she was referred to our hospital for surgical treatment at three months after the rupture of the tumor. At that time, she had no abdominal symptoms and the tumor was not palpable. The CT at our hospital showed the cystic tumor was 4 cm in diameter, which protruded from pancreatic tail and was distant from the main pancreatic duct, and the fluid around the tumor had disappeared (Figure 2). From these findings, an elective laparoscopic enucleation of the tumor was proposed. Since there seemed to be no peritoneal dissemination from the result of laparoscopic exploration, laparoscopic enucleation was performed by using five trocars under pneumoperitoneum. The pancreatic parenchyma was divided with the laparoscopic coagulating shears (Figure 3). Duration of operation was 126 minutes and with little blood loss. Because the content of amylase in the fluid from the drain placed at the cut surface of the pancreas was 9710 IU/L on postoperative day 3, the postoperative pancreatic fistula was confirmed according to the international definition [8]. However, because the volume of the fluid from drain was very small, the drain was removed on postoperative day 4. Except for the pancreatic fistula of grade A [8], the postoperative course was uneventful and the patient was discharged on postoperative day 7. Pathologically, the tumor was diagnosed as SPT of the pancreas. The negative surgical margin and the rupture of the capsule of tumor were verified by microscopic examination (Figure 4). She survived without recurrence of disease for two years after the operation.

## 3. Discussion

It is well known that SPT usually shows no characteristic clinical presentation, and the main nonspecific symptoms are abdominal discomfort or abdominal pain [2–4, 9–12]. In the present case, tumor was discovered by acute abdominal pain due to rupture of the tumor and hemoperitoneum. The incidence of the rupture of SPT was reported in 2.7% of 292 cases [13]. Regarding rupture of SPT, blunt abdominal trauma was the most common [5–7] and according to a review article, 3% of 718 cases of SPT were discovered after the trauma [3]. Other cases were considered to be spontaneous rupture and it was estimated to occur in approximately 1% of all SPT [13–15].

Since the cystic part of SPT consisted of the degeneration after the intramural hemorrhage [7, 9, 13, 16], SPT had a natural tendency to hemorrhage inside the tumor. Therefore, the spontaneous rupture of SPT was considered to result from both abrupt massive hemorrhage and increase of the pressure in the tumor. Panieri and colleagues reported a case of the major bleed into the lesser sac causing the rupture of SPT [16], and the enhanced CT also revealed a massive bleeding around the pancreatic tumor when our patient felt acute abdominal pain. Moreover, the tumor of the present case seemed to be slightly larger at the moment of rupture than three months later. When the abrupt hemorrhage occurred in the abdomen, emergency laparotomy was usually indicated. However, in the present case, the hemorrhage stopped with conservative therapy and allowed elective surgical treatment.

Surgical resection is the only treatment to cure SPT generally assuring a good prognosis, as reflected in the overall 5-year survival rate of approximately 95% [2, 3, 12]. Most cases of SPT have previously undergone the standard pancreatic resections such as pancreatoduodenectomy and distal pancreatectomy, because the tumors were relatively large and misdiagnosed as malignant disease [9, 11]. However, the surgical procedure indicated for SPT still remains controversial. Namely, taking into account the character of SPT, if at all possible the surgical procedures preserving pancreatic parenchyma and function should be selected. Butte and colleagues [12] described microscopic margins that were not considered a strong prognostic factor for the disease recurrence in SPT. Therefore, either partial resection or enucleation might be indicated for the small SPT, especially those localized in the periphery of the pancreas [2, 4, 11, 17, 18]. Actually, local resections and enucleations were performed in approximately 30% of SPT according to the recent literature [2, 3, 19, 20].

On the other hand, there was a report that enucleation for the pancreatic tumor had a high risk of pancreatic fistula [9]. Although postoperative pancreatic fistula was reported in 38% of sixty-one enucleation cases of the pancreatic tumor, it was considered a safe and effective procedure [17, 21]. Li and colleagues [19] recommended a minimized resection such as enucleation and partial resection for SPT, because there were no significant differences in the incidence of postoperative complications and the prognosis between the minimized resection and the standard resection.

In pancreatic surgery, the laparoscopic technique has been increasingly applied in recent years, but it still remains uncommon. Both enucleation and distal pancreatectomy were procedures without reconstruction. Therefore, they were the first to be performed by the laparoscopic approach [21, 22]. These laparoscopic procedures of enucleation and distal pancreatectomy have already been considered to be feasible [20–22] and have shown good indications for the low-grade malignant SPT [11, 21, 22]. It was mentioned that there were no statistically significant differences in the postoperative complications and the prognosis between laparoscopic surgery and ordinary surgery for SPT [20]. With regard to SPT, parenchyma preserving surgical procedure with laparoscopy should be considered if the tumor is small in size and located away from the main pancreatic duct.

There might be another argument in the surgical approach for ruptured SPT. Namely, when the emergency operation was decided just after the rupture of SPT, open surgery might usually be selected. Since acute hemorrhage due to the rupture of SPT was stopped by conservative therapy in the present case, we considered elective laparoscopic surgery. But if the peritoneal dissemination was seen on intraoperative laparoscopic exploration, the conversion to open surgery was considered necessary for curable resection of a metastatic lesion with primary tumor.

Recurrence was reported to occur in 10% to 15% of SPT patients after surgical treatment [2, 4, 10, 12]. Kim and colleagues [11] asserted that the tumor rupture might be the risk factor for recurrence after surgery for SPT. The previous reports showed that peritoneal dissemination of SPT occurred in only few cases even after the rupture [4, 14, 23, 24], although rupture of SPT was expected to be the most predictable factor for the peritoneal dissemination [14, 25]. 

Actually, some case reports indicated that disease free periods of the ruptured cases ranged from 12 to 66 months [5, 6, 15, 25, 26]. On the other hand, Kyokane et al. reported that the dissemination associated with the rupture of SPT was seen in only one case among the six cases of SPT with peritoneal dissemination [14]. Since the rupture of SPT has been quite uncommon, the relationship between rupture of SPT and peritoneal dissemination remains to be resolved.

In conclusion, a quite uncommon case of spontaneous rupture of the solid pseudopapillary tumor of the pancreas has been described. Although emergency laparotomy is usually performed in the case with hemoperitoneum associated with rupture of the pancreatic tumor, we were able to resect SPT completely by elective laparoscopic surgery. SPT is considered to be a low-grade malignancy and a good prognosis is expected after the surgery. However, our patient who is alive without recurrence after surgery for two years should continue to be followed up carefully.
